# Sperm Length Variation as a Predictor of Extrapair Paternity in Passerine Birds

**DOI:** 10.1371/journal.pone.0013456

**Published:** 2010-10-18

**Authors:** Jan T. Lifjeld, Terje Laskemoen, Oddmund Kleven, Tomas Albrecht, Raleigh J. Robertson

**Affiliations:** 1 National Centre for Biosystematics, Natural History Museum, University of Oslo, Oslo, Norway; 2 Department of Population Biology, Institute of Vertebrate Biology, Academy of Sciences of the Czech Republic, and Department of Zoology, Faculty of Science, Charles University in Prague, Brno, Czech Republic; 3 Department of Biology, Queen's University, Kingston, Ontario, Canada; University of Exeter, United Kingdom

## Abstract

**Background:**

The rate of extrapair paternity is a commonly used index for the risk of sperm competition in birds, but paternity data exist for only a few percent of the approximately 10400 extant species. As paternity analyses require extensive field sampling and costly lab work, species coverage in this field will probably not improve much in the foreseeable future. Recent findings from passerine birds, which constitute the largest avian order (∼5 900 species), suggest that sperm phenotypes carry a signature of sperm competition. Here we examine how well standardized measures of sperm length variation can predict the rate of extrapair paternity in passerine birds.

**Methodology/Principal Findings:**

We collected sperm samples from 55 passerine species in Canada and Europe for which extrapair paternity rates were already available from either the same (*n* = 24) or a different (*n* = 31) study population. We measured the total length of individual spermatozoa and found that both the coefficient of between-male variation (CV_bm_) and within-male variation (CV_wm_) in sperm length were strong predictors of the rate of extrapair paternity, explaining as much as 65% and 58%, respectively, of the variation in extrapair paternity among species. However, only the CV_bm_ predictor was independent of phylogeny, which implies that it can readily be converted into a currency of extrapair paternity without the need for phylogenetic correction.

**Conclusion/Significance:**

We propose the CV_bm_ index as an alternative measure to extrapair paternity for passerine birds. Given the ease of sperm extraction from male birds in breeding condition, and a modest number of sampled males required for a robust estimate, this new index holds a great potential for mapping the risk of sperm competition across a wide range of passerine birds.

## Introduction

Sperm competition occurs when females mate promiscuously and sperm from two or more males compete for the fertilization of the same eggs [1], [2]. Sperm competition plays a significant role in the mating system of many animals and selects for a wide variety of adaptations in morphology, physiology and behaviour in the two sexes [3]. It also shapes the morphology and behaviour of sperm cells [4]. A major challenge for evolutionary biologists is to describe the variation in sperm competition levels among species and explain the causes and consequences of this variation. However, there is no consensus of how sperm competition should be representatively measured. Detailed observations of mating behaviour and molecular analyses of paternity patterns might provide useful information about the risk and outcome of sperm competition, but such data are relatively costly to obtain and therefore not available for very many species. More indirect variables, like relative testis size, are easier to obtain from most species, but may be rather crude measures with large sampling errors [5]. Future progress in the study of the evolutionary role of sperm competition therefore hinges on better measures of sperm competition and a broader coverage of species.

Birds display large contrasts in sperm competition levels, as indicated by the variable rates of extrapair paternity among species [6]. Contrasts are especially large in passerine birds, which is the most speciose avian order, encompassing about 5900 of 10400 extant species [7]. Despite extensive paternity analyses in more than a hundred bird species so far (e.g. the Griffith et al. review [6] listed 80 passerine and 51 non-passerine species), it largely remains a puzzle why extrapair paternity rates vary so much, even among closely related species with apparently very similar breeding ecology [6], [8], [9]. Yet, information is still missing for the great majority of bird species, and especially so for those in the tropics [10]. A major constraint for paternity data is that they require extensive sampling of offspring and their parents [6], which can be logistically challenging in many species. Besides, paternity analyses are resource demanding in terms of equipment, consumables and trained personnel. An alternative sperm competition index is relative testis size, as sperm competition seems to select for increased sperm production rates [11]. Testis size data are available for a much longer list of species [12], [13], but a recent critical review [5] concluded that the quality of such data is rather poor, as values from only 11% of 1 044 avian species were considered reliable.

Recently, there has been a common recognition of the role of sperm competition for the evolutionary diversification of sperm cells [4]. Comparative analyses of passerine birds have indicated that sperm competition selects for both longer and faster-swimming sperm [14], [15], [16]. At the intraspecific level, sperm competition is associated with low between-male variation in sperm length [17], [18], [19]. This is consistent with the hypothesis that sperm competition acts as a stabilizing selection pressure reducing the additive genetic variance for sperm length [20], and favouring males with sperm lengths close to a species-specific optimum. Sperm competition is also associated with reduced within-male variation in sperm length in passerine birds [18], [21]. Sperm competition not only selects for larger testes [11], but also alters the architecture and proportion of sperm producing tissue within the testes [22]. In particular, the diameter of the seminiferous tubules in the testes, i.e. where spermatozoa are formed, was positively related to the average sperm length across 20 species of Icteridae [22]. It is therefore a possibility, that sperm competition leads to more similar-sized seminiferous tubules, and hence more similar-sized sperm, within individual males. In effect, it seems that sperm competition leads to stronger developmental quality control in the sperm production apparatus [17], [21], [23]. Spermatogenesis is under complex developmental control within the diploid male [24], and genetic factors like inbreeding, that reduce developmental homeostasis [25], can lead to production of more heterogeneous spermatozoa within ejaculates [26], [27].

Here we investigate how well sperm length data can predict the rate of extrapair paternity in a given population, and hence can be used as an alternative proxy for sperm competition in passerine birds. Our predictor variables were mean sperm length, within-male variation and between-male variation in sperm length. The primary data set contained 24 species for which sperm length and paternity data originated from the same study populations. This requirement is important since extrapair paternity rates can vary geographically within a species [9]. We also examined the same variables in an expanded data set, including 31 more species in which sperm and paternity data came from different study populations. We analysed the predictive power of the three sperm variables in phylogenetically controlled analyses, where we could also estimate the degree of phylogenetic dependence in the associations. Ideal predictor variables should directly reflect the frequency of extrapair paternity without the need for phylogenetic correction. For comparison, we also tested the predictive power of relative testis size from a subset of data extracted from the literature.

## Materials and Methods

### Data collection and preparation

Data on extrapair paternity (proportion of extrapair young) were collected from articles in peer-review journals, with the exception of four studies currently under preparation for publication (see full reference list in Table S1). All paternity studies were considered methodologically robust with respect to analysis techniques (DNA fingerprinting or microsatellites). Data on relative testis size were extracted from Pitcher et al. [13]. Sperm length data originate from sperm samples collected by us in the field in Canada and Europe, fixed in 5% formalin solution, and photographed and measured in a digital light microscopy system according to a standard protocol. Details of field sampling procedures and sperm length measurements are presented elsewhere [15], [18]. Basically, we measured the total length (±0.1 µm) of 10 sperm cells per male, and in a minimum of four males per species (median  = 10). All sperm measurements were conducted by one person only. As a standardized measure of variation, we used the coefficient of variation (CV  =  SD/mean×100), denoted as CV_bm_ for the between-male CV in mean sperm length and CV_wm_ for the mean within-male CV in sperm length. As CV will be underestimated for small sample sizes, we corrected CV_bm_ according to the formula: Adjusted CV_bm_ =  (1+1/4*n*) × CV_bm_ [28]. Descriptive statistics of sperm length and its variation are given for each species in Table S1.

Our field work was conducted in adherence to the Norwegian regulations for the use of animals in research and approved by the Canadian Wildlife Service (permit no. CA 0155) and Queen's University Animal Care Committee (protocol no. Robertson-2005-014-R1).

### Phylogeny of the study species

Comparative analyses need to take into account that species are not statistically independent data points, and potential effects due to shared ancestry must be controlled for. We therefore needed a phylogeny for our study species. However, a consensus phylogeny for passerine birds does not yet exist, and there is much debate about the topology of the tree, especially at the basal branches. We therefore decided to construct the phylogeny from the most recent molecular phylogenies that are based on multiple genetic markers and more comprehensive taxon sampling. Our argumentation below refers mostly to the phylogenetic relationships at the taxonomic level of the family (family affiliation for each species is indicated in Table S1).

**Figure 1:**
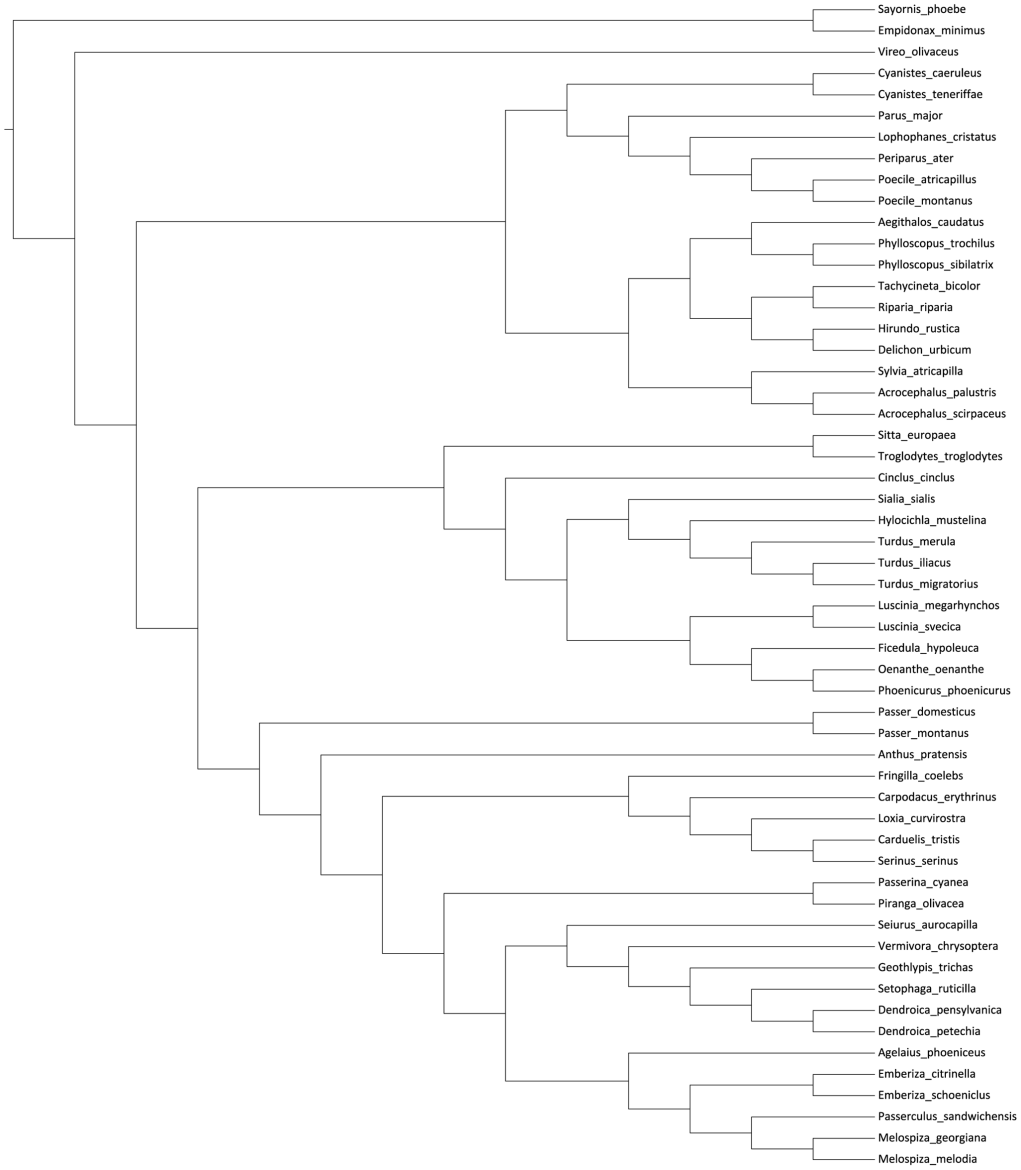
Phylogeny of study species. The figure illustrates the phylogenetic relationships among the 55 study species. The tree was derived from the most recent advances in avian molecular phylogenetics, with emphasis on studies using multiple genes and broad taxonomic coverage. Explanations and references to the different nodes are given in Materials and Methods.

Our phylogeny of the 55 study species is presented in Figure 1. All but two species (Tyrannidae) belong to the oscine passerines (suborder Passeri) whose phylogenetic structure at the deeper nodes is poorly resolved. Here we follow the topology described by Treplin et al. [29] for the relationships among the superfamilies Corvoidea, Sylvioidea, Muscicapoidea and Passeroidea, with Corvoidea (Vireonidae, 1 species) placed basally to the three others. This is consistent with Sibley and Ahlquist's [30] classification of parvorders Corvida and Passerida, respectively. Treplin et al. [29] resolved the shallow relationships among the latter three superfamilies with a combination of four nuclear loci, and found evidence for Sylvioidea as the older clade, and Muscicapoidea and Passeroidea as sister groups. Within Sylvioidea, the Paridae family is a sister to the rest of the Sylvioidea [29]. The phylogenetic relationships among the seven Paridae species in our data set follow Gill et al. [31], with the split between the two *Cyanistes* species as suggested by Salzburger et al. [32] and Kvist et al. [33]. The phylogenetic relationships among the 10 other species belonging to Sylvioidea follow Alström et al. [34] with Aegithalidae (1 sp.) and Phylloscopidae (2 spp.) as sister taxa, which, together the Hirundinidae (4spp.), form a sister clade to the Sylviidae (1 sp.) and Acrocephalidae (2 spp.). The phylogenetic relationships among the Hirundinidae species follows Sheldon et al. [35].

The phylogenetic position of Sittidae and Troglodytidae is enigmatic, but we place them basally within the Muscicapoidea, consistent with the suggestions of Jønsson and Fjeldså [36] and Treplin et al. [29]. Among the remaining species in this superfamily, Cinclidae (1 sp.) is placed as a sister taxon to Turdidae and Muscicapidae, consistent with the results of Jønsson and Fjeldså [36] and Treplin et al. [29]. The phylogenetic relationships within Turdidae (5 spp.) follow Voelker and Klicka [37] for the genera and Voelker et al. [38] for the three *Turdus* species. The relationships among the Muscicapidae (5 spp.) follow Zuccon and Ericson [39] and Sangster et al. [40].

The phylogenetic relationships among families within in the Passeroidea is still poorly resolved, and contrasting topologies exist among several recent studies. Our basal branching of Passeridae, Motacillidae and Fringillidae follows Treplin et al. [29], although Jønsson and Fjeldså [36] indicated an unresolved polytomy for these groups. The phylogeny of the five species of Fringillidae follows Nguembock et al. [41]. The phylogenetic positions of the families of Cardinalidae, Emberizidae, Icteridae and Parulidae are currently unresolved [36], [42], [43], but there seems to be stronger support for Cardinalidae as a basal branch in this clade [36], [43] and Parulidae as a sister group to the Emberizidae and Icteridae [42], [43]. The Parulidae phylogeny (6 spp.) is derived from Lovette et al. [44]. The phylogenetic relationships within the Emberizidae (5 spp.) follow Jønsson and Fjeldså [36] and Alström et al. [42].

### Statistical analyses

In the comparative analyses we applied a generalized least squares regression method in a phylogenetic framework [45], [46] with the phylogeny of species shown in Fig. 1. Constant branch lengths were assumed. We made both univariate and multivariate regressions of the three sperm length variables (total length, CV_bm_ and CV_wm_) on the proportion of extrapair paternity, to assess and compare their predictive power. To improve normality, all three sperm length variables were log transformed and the proportions of extrapair young arcsine-squareroot transformed. The slopes were tested against the prediction of 0 using a *t*-test. For each test, an index of phylogenetic dependence, λ, was estimated, with values ranging between 0 (phylogenetic independence) and 1 (complete phylogenetic dependence), and tested with a likelihood ratio test against models with λ values set at 0 and 1. The analyses were performed in R [47] using the package APE [48] and a script provided by R. P. Freckleton, Department of Animal and Plant Sciences, The University of Sheffield.

## Results

In the primary data set of 24 species, the rate of extrapair pair paternity was positively correlated with mean sperm length, and negatively correlated with both CV_wm_ and CV_bm_ (Table 1A). The association was strongest for CV_bm_, which explained 65% of the variation in extrapair paternity rates. As judged from the λ-values, the association with CV_bm_ was not influenced by phylogeny (λ≈0), in contrast to those of the two other sperm variables which had λ-values not significantly different from 1. When all three sperm variables were included simultaneously in the regression analysis, CV_bm_ remained the strongest predictor, but both sperm length and CV_wm_ still had significant partial effects (Table 1B). Overall, the three variables explained 72% of the total variance in extrapair paternity and λ was not significantly different from 0. Notably, the partial effect of sperm length became negative in the multivariate analysis.

**Table 1:** Generalised least squares regression analyses of the predictive effects of sperm length traits on the rate of extrapair paternity.

Sperm trait	Slope ± SE	*t*-value	*P*	λ	*r* ^2^
**A** 24 species (same study population), separate regressions					
Total length	0.47±0.17	2.76	0.011	0.80^0.041, 0.29^	0.26
CV_wm_	−0.96±0.17	−5.49	<0.001	0.90^0.002, 0.46^	0.58
CV_bm_	−1.07±0.17	−6.32	<0.001	<0.001^1, <0.008^	0.65
**B** 24 species (same study population), multiple regression					
Total length	−0.45±0.15	−3.03	0.007		
CV_wm_	−0.63±0.29	−2.20	0.040		
CV_bm_	−1.01±0.21	−4.78	<0.001		
Combined				<0.001^1, 0.001^	0.72
**C** 55 species (same or different population), separate regressions					
Total length	0.33±0.16	2.07	0.043	0.81^0.002, 0.027^	0.07
CV_wm_	−0.87±0.18	−4.85	<0.001	0.89^<0.001, 0.14^	0.31
CV_bm_	−0.86±0.14	−5.96	<0.001	0.88^<0.001, 0.14^	0.40
**D** 55 species (same or different population), multiple regression					
Total length	−0.16±0.16	−0.97	0.33		
CV_wm_	−0.56±0.24	−2.37	0.022		
CV_bm_	−0.64±0.17	−3.75	<0.001		
Combined				0.91^<0.001, 0.22^	0.44

CV_wm_ is the average coefficient of within-male variation in sperm length. CV_bm_ is the coefficient of between-male variation is sperm length, adjusted for sample size (see Methods). The statistical analyses were based on transformed variables to approach normality (arcsine square-root for the proportion of extrapair young, log_10_ for sperm length, CV_wm_ and CV_bm_). Slopes were tested against the prediction of 0 using a *t*-test. The λ-values express the degree of phylogenetic dependence of the associations, and the two superscripts indicate *P*-values of likelihood ratio tests of λ against models of λ = 0 (no phylogenetic dependence) and λ = 1 (full phylogenetic dependence), respectively. *r* ^2^ values indicate the proportion of total variance explained.

When we included 31 additional species with paternity data from a population other than that from which we collected sperm samples, both the univariate (Table 1C) and the multivariate (Table 1D) regressions revealed weaker associations. This is as expected when sperm competition varies among populations of the same species. Furthermore, the effect of CV_bm_ showed stronger phylogenetic dependence in the combined data set, which would indicate that the match with extrapair paternity was particularly poor for some of these additional species. This could arise from less representative extrapair paternity rates in some species, for example due to larger geographic differences in some species than in others, or small sample sizes.

**Figure 2:**
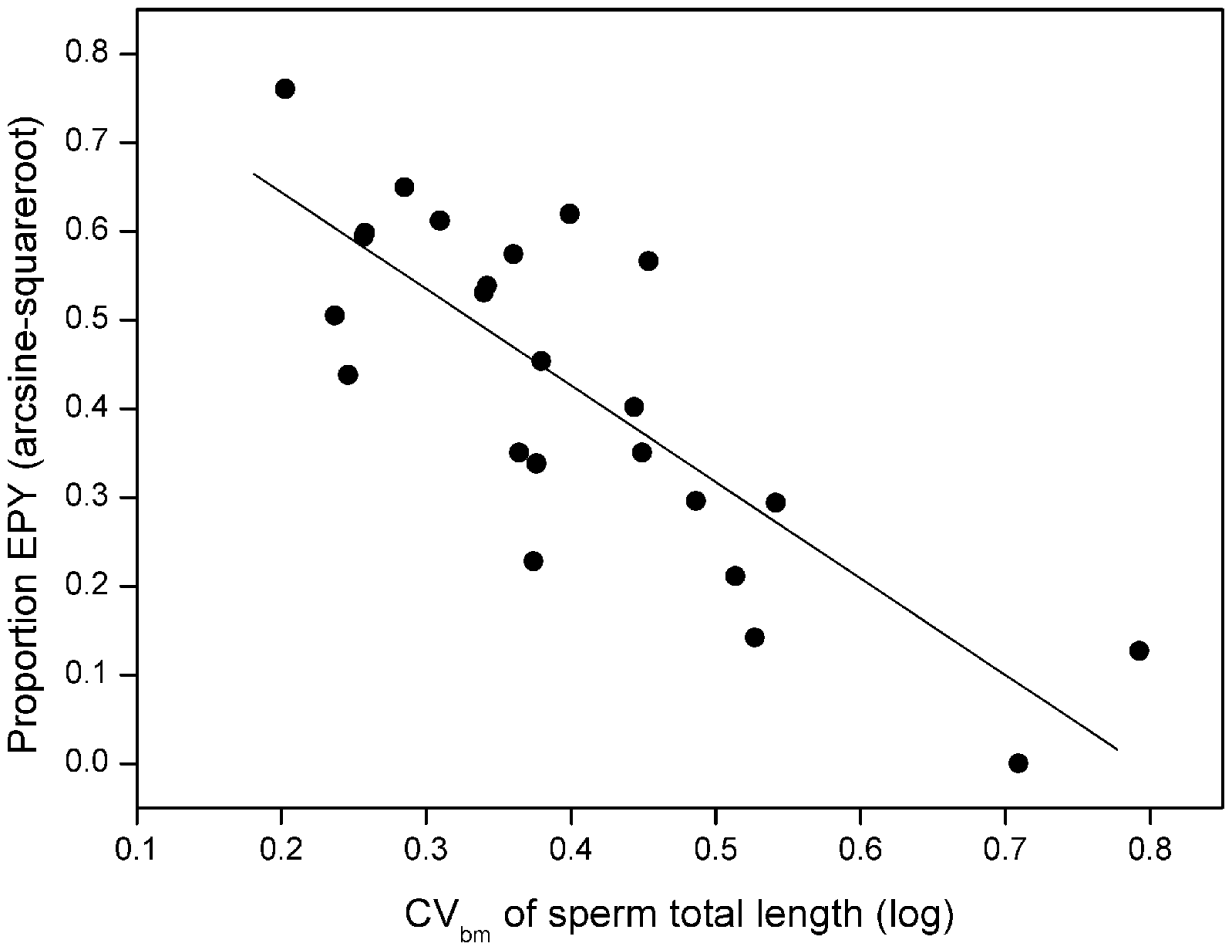
Relationship between extrapair paternity and between-male variation in sperm length. The figure illustrates the linear regression of the proportion of extrapair young on the coefficient of between-male variation in sperm length (CV_bm_) for 24 passerine species in which paternity and sperm data originated from the same study population (raw data given in Table S1). Note that transformed values are used. The regression line (y = 0.8614–1.0882×; *r* ^2^ = 0.66, *P*<0.001) was used to calculate predicted extrapair paternity rates from CV_bm_ values for all 55 species in the data set (see Table S1).

**Figure 3:**
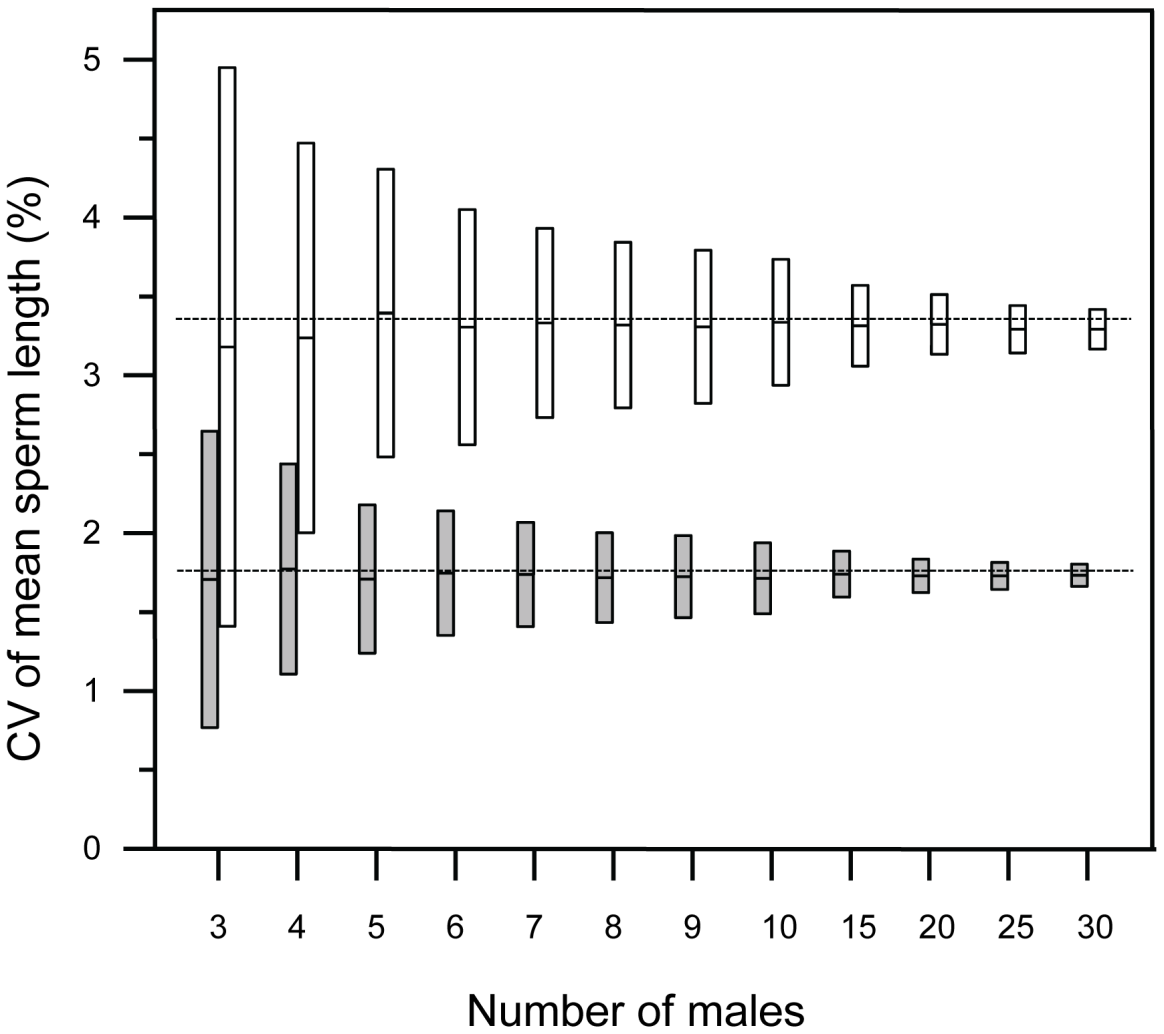
The effect of sample size on the coefficient of variation in male sperm length. The figure shows how the spread of calculated CV_bm_ values (the coefficient of variation in mean sperm length) changes as a function of the number of males sampled. A resampling procedure was performed on two data sets; one from 35 male common redstarts *Phoenicurus phoenicurus* (open boxes) and one from 46 male tree swallows *Tachycineta bicolor* (grey boxes). CV_bm_ was calculated from 1000 random samples (with replacement) for each sample size of 3 to 30 males. Boxes indicate 95% confidence intervals of the mean. Stippled lines mark the CV_bm_ values calculated for all males in the two samples.

The close association between CV_bm_ and extrapair paternity rate in the primary data set of 24 species is illustrated in Figure 2. The lack of phylogenetic signal in this relationship (Table 1A) implies that species can be treated as independent data points without phylogenetic correction. The linear regression line can thus be used as a formula to calculate an expected extrapair paternity rate in species where only data on CV_bm_ of sperm length exist. In Table S1 we give such predictive estimates of extrapair paternity rates along with observed extrapair paternity rates. Clearly, the precision of such estimates depends on the accuracy of the CV_bm_-value for a given species, which is influenced by the number of males sampled. We used a resampling procedure to illustrate how the variation in CV_bm_-estimates diminishes as the number of sampled males increases, and two examples are given in Figure 3. The two species differ markedly in extrapair paternity rates and their overlap in resampled CV_bm_-values was minute even at sample sizes of only 4–5 males. The simulations also confirmed that the use of the sample-size correction for the coefficient of variation (see Materials and Methods) effectively counteracted any systematic underestimation of CV_bm_-values for smaller sample sizes.

Relative testis size proved to be a poor predictor of extrapair paternity. Data on relative testis size [12] were available for 45 of the 55 species included in our analyses (Table S1), and this variable explained only 15% of the variation in extrapair paternity (Generalized least squares regression: slope  = 0.26±0.10, *t* = 2.71, *P* = 0.010, λ = 0.69; significantly different from both λ = 1 [*P* = 0.010] and λ = 0 [*P* = 0.017], *r* ^2^ = 0.15).

## Discussion

Several recent comparative analyses of sperm morphometrics in passerine birds [15], [17], [18], [21] have documented the same qualitative relationships with indices of sperm competition as we have shown here; that higher risk of sperm competition is associated with longer sperm and reduced between-male and within-male variation in sperm length. With an expanded data set, we have been able to compare the strength of these relationships and their degree of phylogenetic dependence. The between-male variation in sperm length (CV_bm_) stands out as an exceptionally strong correlate of the rate of extrapair paternity, and the linear relationship between the two variables (transformed values) showed no phylogenetic bias (λ≈0). Very similar test results were also found for this variable by Calhim et al. [17] and Kleven et al. [18] with smaller data sets. We have also confirmed that relative testis size is a rather poor predictor of extrapair paternity.

We anticipate two major advantages with this new index based on sperm length variation. First, its strong negative correlation with extrapair paternity suggests that it is a meaningful proxy for sperm competition, perhaps reflecting the strength of sperm competition as an evolutionary force of stabilizing selection. A quantitative genetics study in zebra finches (*Taeniopygia guttata*) documented strong direct and indirect genetic effects on sperm phenotypes, as well as large between-male variation in sperm size and its linear components [20]. This variation may seem surprising, as sperm should always be under strong selection to fertilize eggs. However, extrapair paternity is actually quite infrequent in this species [49], which should imply relaxed selection on sperm phenotypes from sperm competition [20]. A prediction of this stabilizing selection hypothesis is that the absolute difference of a male's sperm length from the population mean should be negatively correlated with his success in sperm competition, but we are not aware of any empirical support for this prediction yet. It is difficult to know whether the CV_bm_ index performs better or worse than extrapair paternity as a proxy for sperm competition, because sperm competition itself has no operational currency. Due to high heritability and additive genetic variance in sperm size [50], the CV_bm_ index might be regarded as a more intrinsic, long-term signature of sperm competition that should be less prone to temporal or spatial fluctuations than is the case for extrapair paternity rates, which is a behavioral trait that can fluctuate considerably between years and habitats [51], [52]. Extrapair paternity rates may not correctly picture the instant level of sperm competition either, because sperm competition may well occur without resulting in extrapair paternity [53]. There can be biased outcomes of sperm competition, as modeled in “fair” versus “loaded raffles” [54], and such outcomes can vary considerably at the individual level [55]. But at the species or population level, we assume that extrapair paternity rates, expressed as the proportion of extrapair offspring in the population, should fairly well reflect the relative numbers of extrapair to withinpair sperm cells in direct competition for fertilizations over the population as a whole. We have therefore also deliberately chosen to use the proportion of extrapair young, instead of the proportion of broods with (one or more) extrapair offspring, (see e.g. [56]), because the latter measure will inevitably be biased by clutch size differences among species. If the CV_bm_ index is a more long-term, evolutionary signature of sperm competition, and extrapair paternity a more contemporary one that is more sensitive to ecological conditions and fluctuations, the two indices of sperm competition may actually complement each other in comparative analyses. At the same time, one should keep in mind that sperm morphology can also show rapid evolutionary responses to selection, as evidenced in recent artificial selection experiments [57], and give rise to intraspecific, geographic variation in sperm size. This has recently been shown in red-winged blackbirds (*Agelaius phoeniceus*) [58]. It would be very interesting to test whether a negative correlation between the CV_bm_ metric and extrapair paternity also exists at the intraspecific, interpopulational level, as we have documented here for the between-species level. We therefore encourage studies of geographical variation in sperm morphology and variability within passerine species with known population differences in extrapair paternity rates.

Another major advantage with the CV_bm_ index is that it is logistically and analytically simple. Sperm samples are easy to collect from breeding males of most passerine species [15], [18] and the birds can be released unharmed with no ethical concerns beyond the catching and handling of wild birds during the breeding season. This is in contrast to the alternatives which either requires sacrificing a number of individuals (testis size) or extensive blood sampling of parents and their offspring for parentage analysis. Moreover, the measurement of sperm length in the linear dimension is straightforward and accurate with low measurement errors and high repeatability of average sperm length for individual males [18], [59], [60]. This contrasts with a number of methodological difficulties of obtaining representative measures of species-specific testis size, which is largely due to the fact that testes show strong seasonal and age-dependent size variation in birds [5], [61], [62]. The precise measure of sperm lengths in the range of 40–300 µm requires a good microscope, preferentially with digital imaging and morphometrics software, which is standard equipment in most biological research institutions.

The index of within-male variation in sperm length (CV_wm_) also seems a strong correlate of extrapair paternity, but it is potentially influenced by additional environmental factors associated with the development and growth of the testes. Such factors may be more influential in some species than in others, causing a phylogenetic signal in its relationship with extrapair paternity. The claim of Immler et al. [21], that the negative association between extrapair paternity and within-male variation in sperm length has a phylogenetic scaling parameter (λ) close to 0, does not seem to hold true for larger data sets. Hence, the CV_wm_ metric seems less suitable as general index of extrapair paternity, since its scaling effect can only be assessed in a phylogenetic framework.

Although our analysis has indicated a close association between the CV_bm_ index and extrapair paternity, with no evidence of a phylogenetic bias, we must emphasize that our selection of species is rather restricted and concentrated on the Passerida clade (*sensu* Sibley and Ahlquist [30]) within the order Passeriformes. Hence our findings should be tested across a wider taxonomic range to infer generality. It is also important to stress that the lack of phylogenetic bias in the relationship between the two proxies for sperm competition, CV_bm_ and extrapair paternity, does not preclude the need for control of phylogenetic effects in comparative analyses of sperm competition. Obviously, there is a strong phylogenetic signal in the variation of extrapair paternity rates across species [8], but the CV_bm_ index seems to reflect this variation equally well over the entire phylogeny.

We conclude that the CV_bm_ index holds a great potential for comparative, as well as between-population, analysis of sperm competition in passerine birds. Whether it can be applied to other taxonomic groups remains to be studied, but circumstantial evidence suggests that a negative relationship between intraspecific sperm length variation and sperm competition risk also exist in mammals [63], e.g. shrews [64], murine rodents [65] and hominids [66].

## Supporting Information

Table S1 Sperm length characteristics, relative testis size, predicted proportions of extrapair young from the CVbm index, and observed proportions of extrapair young in 55 passerine species.
(0.15 MB DOC)
