# Supplementary material for: Sperm Length Variation as a Predictor of Extrapair Paternity in Passerine Birds
Source: PLoS One. 2010 Oct 18;5(10):e13456. doi: 10.1371/journal.pone.0013456 (PMC2956655; doi:10.1371/journal.pone.0013456)
Supplement: Table S1 — Sperm length characteristics, relative testis size, predicted proportions of extrapair young from the CVbm index, and observed proportions of extrapair young in 55 passerine species. (0.15 MB DOC) [file pone.0013456.s001.doc]

**Table S1 Sperm length characteristics, relative testis size, predicted proportions of extrapair young from the CV_bm_ index, and observed proportions of extrapair young in 55 passerine species.**

| Species | Family | Sperm sampling location | Mean sperm length (µm) | SD (µm) | *n* males | CV_wm_ | CV_bm_ | Residual testis size^1^ | Predicted EPY | Observed EPY^2^ | Source of EPY data |
| --- | --- | --- | --- | --- | --- | --- | --- | --- | --- | --- | --- |
| Acrocephalus palustris | Acrocephalidae | Southern Norway | 108.4 | 4.6 | 8 | 2.48 | 4.33 | 0.041962 | 0.028 | 0.031 | Leisler & Wink 2000 |
| Acrocephalus scirpaceus | Acrocephalidae | Southern Norway | 109.2 | 2.3 | 6 | 2.24 | 2.15 | 0.071925 | 0.230 | 0.065 | Davies *et al.* 2003 |
| Aegithalos caudatus | Aegithalidae | Southern Norway | 70.5 | 2.3 | 7 | 2.54 | 3.33 |  | 0.084 | 0.024 | Hatchwell *et al.* 2002 |
| Agelaius phoeniceus | Icteridae | Ontario, Canada | 146.0 | 3.1 | 10 | 1.49 | 2.19 | 0.234426 | 0.223 | **0.256** | Weatherhead & Boag 1995 |
| Anthus pratensis | Motacillidae | Southern Norway | 71.6 | 1.1 | 10 | 1.76 | 1.63 | 0.293241 | 0.346 | 0.270 | Nadvornik 2004 |
| Carduelis tristis | Fringillidae | Ontario, Canada | 197.3 | 3.1 | 4 | 1.16 | 1.67 | 0.35999 | 0.335 | 0.143 | Gissing *et al.* 1998 |
| Carpodacus erythrinus | Fringillidae | Czech Republic | 275.4 | 4.7 | 11 | 1.14 | 1.76 | 0.114979 | 0.313 | **0.180** | Albrecht *et al.* 2007 |
| Cinclus cinclus | Cinclidae | Southern Norway | 43.5 | 2.6 | 6 | 3.08 | 6.20 |  | 0.000 | **0.016** | Øigarden *et al.* 2010 |
| Cyanistes caeruleus | Paridae | Southern Norway | 104.6 | 2.5 | 21 | 2.13 | 2.38 | 0.37075 | 0.191 | **0.110** | Krokene & Lifjeld 2000;  Johannessen *et al.* 2005 |
| Cyanistes teneriffae | Paridae | Tenerife, Spain | 114.4 | 3.1 | 9 | 1.67 | 2.78 |  | 0.137 | **0.153** | Own unpublished data |
| Delichon urbicum | Hirundinidae | Southern Norway | 98.8 | 2.3 | 8 | 2.19 | 2.40 | 0.10431 | 0.188 | **0.192** | Whittingham & Lifjeld 1995 |
| Dendroica pensylvanica | Parulidae | Ontario, Canada | 243.3 | 4.2 | 10 | 1.32 | 1.78 | -0.14178 | 0.308 | 0.474 | Byers *et al.* 2004 |
| Dendroica petechia | Parulidae | Ontario, Canada | 271.8 | 5.1 | 10 | 1.35 | 1.93 | 0.185578 | 0.274 | **0.366** | Yezerinac *et al.* 1995 |
| Emberiza citrinella | Emberizidae | Southern Norway | 130.2 | 2.1 | 10 | 1.36 | 1.62 | 0.154534 | 0.350 | 0.374 | Sundberg & Dixon 1996 |
| Emberiza schoeniclus | Emberizidae | Southern Norway | 278.5 | 6.2 | 10 | 1.32 | 2.29 | 0.584133 | 0.205 | **0.295** | Kleven & Lifjeld 2005 |
| Empidonax minimus | Tyrannidae | Ontario, Canada | 52.0 | 1.3 | 10 | 2.71 | 2.51 | 0.205056 | 0.172 | **0.337** | Tarof *et al.* 2005 |
| Ficedula hypoleuca | Muscicapidae | Southern Norway | 101.6 | 3.2 | 10 | 2.14 | 3.26 | -0.50211 | 0.089 | **0.044** | Lifjeld *et al.* 1991 |
| Fringilla coelebs | Fringillidae | Southern Norway | 259.0 | 4.8 | 10 | 1.20 | 1.90 | 0.469159 | 0.280 | 0.170 | Sheldon & Burke 1994 |
| Geothlypis trichas | Parulidae | Ontario, Canada | 167.1 | 4.1 | 10 | 1.52 | 2.55 | 0.253536 | 0.166 | 0.203 | Thusius *et al.* 2001;  Garvin *et al.* 2006 |
| Hirundo rustica | Hirundinidae | Ontario, Canada | 87.9 | 2.5 | 87 | 1.74 | 2.84 | 0.279453 | 0.129 | **0.288** | Kleven *et al.* 2005 |
| Hylocichla mustelina | Turdidae | Ontario, Canada | 81.9 | 1.7 | 10 | 1.69 | 2.18 |  | 0.224 | 0.205 | Evans *et al.* 2008;  Evans *et al.* 2009 |
| Lophophanes cristatus | Paridae | Southern Norway | 85.0 | 2.2 | 8 | 2.47 | 2.63 |  | 0.155 | 0.110 | Lens *et al.* 1997 |
| Loxia curvirostra | Fringillidae | Southern Norway | 99.2 | 4.9 | 7 | 3.64 | 5.12 | -0.23919 | 0.008 | **0.000** | Kleven *et al.* 2008 |
| Luscinia megarhynchos | Muscicapidae | Czech Republic | 279.9 | 7.8 | 6 | 1.31 | 2.92 |  | 0.121 | 0.075 | Amrheim 2004 |
| Luscinia svecica | Muscicapidae | Southern Norway | 212.4 | 4.6 | 10 | 1.26 | 2.20 | 0.183491 | 0.221 | **0.263** | Johnsen & Lifjeld 2003 |
| Melospiza georgiana | Emberizidae | Ontario, Canada | 237.4 | 1.7 | 18 | 0.98 | 1.13 | 0.62694 | 0.517 | 0.209 | Olsen *et al.* 2008 |
| Melospiza melodia | Emberizidae | Ontario, Canada | 224.5 | 5.1 | 16 | 1.59 | 2.37 | 0.039446 | 0.193 | **0.051** | E. MacDougall-Shackleton  unpublished data |
| Oenanthe oenanthe | Muscicapidae | Southern Norway | 187.0 | 4.3 | 4 | 1.40 | 2.46 | 0.541636 | 0.179 | 0.205 | Currie *et al.* 1998;  Kudernatsch *et al.* 2010 |
| Parus major | Paridae | Southern Norway | 98.9 | 3.0 | 10 | 2.34 | 3.06 | -0.11233 | 0.106 | **0.085** | Johannessen *et al.* 2005 |
| Passer domesticus | Passeridae | Southern Norway | 99.0 | 2.4 | 10 | 1.93 | 2.51 | 0.320797 | 0.171 | 0.124 | Wetton & Parkin 1991;  Stewart *et al.* 2006 |
| Passer montanus | Passeridae | Southern Norway | 94.2 | 2.3 | 10 | 1.67 | 2.48 | 0.52716 | 0.176 | 0.091 | Cordero *et al.* 2002 |
| Passerculus sandwichensis | Emberizidae | Ontario, Canada | 233.5 | 3.4 | 10 | 1.15 | 1.51 | 0.465323 | 0.382 | 0.405 | Freeman-Gallant 1996;  Freeman-Gallant *et al.* 2005 |
| Passerina cyanea | Cardinalidae | Ontario, Canada | 278.6 | 6.8 | 10 | 1.16 | 2.50 | 0.008703 | 0.172 | 0.349 | Westneat 1990 |
| Periparus ater | Paridae | Germany | 91.7 | 2.4 | 10 | 2.03 | 1.81 | 0.083285 | 0.301 | **0.317** | Schmoll *et al.* 2005 |
| Phoenicurus phoenicurus | Muscicapidae | Southern Norway | 164.3 | 5.5 | 35 | 1.82 | 3.37 | -0.14513 | 0.081 | **0.020** | Kleven *et al.* 2007 |
| Phylloscopus sibilatrix | Phylloscopidae | Southern Norway | 105.8 | 2.6 | 10 | 1.37 | 2.54 | 0.176398 | 0.167 | 0.000 | Gyllensten *et al.* 1990 |
| Phylloscopus trochilus | Phylloscopidae | Southern Norway | 93.5 | 1.9 | 42 | 1.46 | 2.04 | 0.089191 | 0.251 | **0.330** | Bjørnstad & Lifjeld 1997 |
| Piranga olivacea | Cardinalidae | Ontario, Canada | 160.1 | 3.2 | 7 | 1.55 | 2.09 | 0.368931 | 0.242 | 0.167 | Klatt *et al.* 2008 |
| Poecile atricapillus | Paridae | Ontario, Canada | 85.7 | 1.9 | 10 | 2.72 | 2.31 | 0.312132 | 0.201 | **0.118** | Otter *et al.* 1998;  Mennill *et al.* 2004 |
| Poecile montanus | Paridae | Southern Norway | 91.6 | 3.0 | 10 | 2.23 | 3.33 | -0.22384 | 0.084 | 0.106 | Orell *et al.* 1997 |
| Riparia riparia | Hirundinidae | Southern Norway | 123.9 | 3.8 | 31 | 1.96 | 3.09 | -0.06595 | 0.104 | 0.174 | Alves & Bryant 1998;  Augustin *et al.* 2007 |
| Sayornis phoebe | Tyrannidae | Ontario, Canada | 48.0 | 1.3 | 4 | 3.30 | 2.81 |  | 0.133 | **0.118** | Conrad *et al.* 1998 |
| Seiurus aurocapilla | Parulidae | Ontario, Canada | 188.3 | 4.1 | 10 | 1.34 | 2.25 | 0.076157 | 0.212 | 0.278 | Roberts 2005 |
| Serinus serinus | Fringillidae | Czech Republic | 248.0 | 5.3 | 5 | 0.97 | 2.25 |  | 0.212 | 0.094 | Hoi-Leitner *et al.* 1999 |
| Setophaga ruticilla | Parulidae | Ontario, Canada | 248.9 | 4.2 | 10 | 1.33 | 1.73 | 0.431517 | 0.322 | **0.234** | Reudink *et al.* 2009 |
| Sialia sialis | Turdidae | Ontario, Canada | 124.2 | 4.2 | 10 | 3.06 | 3.48 | 0.083716 | 0.072 | **0.084** | Meek *et al.* 1994 |
| Sitta europaea | Sittidae | Southern Norway | 78.0 | 3.0 | 4 | 1.78 | 4.05 |  | 0.040 | 0.096 | Segelbacher *et al.* 2005 |
| Sylvia atricapilla | Sylviidae | Southern Norway | 75.9 | 2.2 | 8 | 1.82 | 2.99 | 0.70637 | 0.114 | 0.148 | G. Segelbacher unpublished data |
| Tachycineta bicolor | Hirundinidae | Ontario, Canada | 236.0 | 3.7 | 46 | 1.22 | 1.59 | 0.586169 | 0.358 | **0.475** | Stapleton *et al.* 2007;  Delmore *et al.* 2008 |
| Troglodytes troglodytes | Troglodytidae | Southern Norway | 88.6 | 1.7 | 10 | 2.30 | 1.96 | -0.12463 | 0.266 | 0.163 | Brommer *et al.* 2007 |
| Turdus iliacus | Turdidae | Southern Norway | 83.1 | 3.3 | 10 | 3.11 | 4.08 |  | 0.038 | 0.132 | Asklund 2008 |
| Turdus merula | Turdidae | Southern Norway | 80.8 | 3.4 | 5 | 1.80 | 4.41 | 0.535684 | 0.026 | 0.186 | J. Rutkowska unpublished data |
| Turdus migratorius | Turdidae | Ontario, Canada | 90.8 | 2.1 | 9 | 1.92 | 2.35 | 0.494169 | 0.195 | 0.481 | Rowe & Weatherhead 2007 |
| Vermivora chrysoptera | Parulidae | Ontario, Canada | 201.8 | 3.6 | 10 | 1.28 | 1.81 | 0.060468 | 0.302 | **0.313** | Vallender *et al.* 2007 |
| Vireo olivaceus | Vireonidae | Ontario, Canada | 82.6 | 1.8 | 5 | 1.90 | 2.28 | 0.414505 | 0.207 | 0.579 | Morton *et al.* 1998 |

^1^ Values taken from Pitcher et al. 2005 (Ref. no. 13).

^2^ Numbers in bold indicate species in which data on EPY (proportion of extrapair young) originate from the same population as the sperm data

**References**

Albrecht, T., Schnitzer, J., Kreisinger, J., Exnerova, A., Bryja, J. & Munclinger, P. 2007 Extrapair paternity and the opportunity for sexual selection in long-distant migratory passerines. Behav. Ecol. 18, 477-486.

Alves, M. A. S. & Bryant, D. M. 1998 Brood parasitism in the sand martin, Riparia riparia: evidence for two parasitic strategies in a colonial passerine. Anim. Behav. 56, 1323-1331.

Amrheim, V. 2004 Singing activity and spatial behaviour as sexually selected traits in the nightingale *Luscinia megarhynchos*. Ph.D. thesis. Basel: University of Basel.

Asklund, T. M. 2008 Factors affecting extra-pair paternity in redwings (Turdus iliacus). M.Sc. thesis. Department of Biology. Trondheim: Norwegian University of Science and Technology.

Augustin, J., Blomqvist, D., Szep, T., Szabo, Z. D. & Wagner, R. H. 2007 No evidence of genetic benefits from extra-pair fertilisations in female sand martins (*Riparia riparia*). J. Ornithol. 148, 189-198.

Bjørnstad, G. & Lifjeld, J. T. 1997 High frequency of extra-pair paternity in a dense and synchronous population of willow warblers *Phylloscopus trochilus*. J. Avian Biol. 28, 319-324.

Brommer, J. E., Korsten, P., Bouwman, K. A., Berg, M. L. & Komdeur, J. 2007 Is extrapair mating random? On the probability distribution of extrapair young in avian broods. Behav. Ecol. 18, 895-904.

Byers, B. E., Mays, H. L., Stewart, I. R. K. & Westneat, D. F. 2004 Extrapair paternity increases variability in male reproductive success in the chestnut-sided warbler (*Dendroica pensylvanica*), a socially monogamous songbird. Auk 121, 788-795.

Conrad, K. F., Robertson, R. J. & Boag, P. T. 1998 Frequency of extrapair young increases in second broods of eastern phoebes. Auk 115, 497-502.

Cordero, P. J., Heeb, P., Wetton, J. H. & Parkin, D. T. 2002 Extra-pair fertilizations in tree sparrows *Passer montanus*. Ibis 144, E67-E72.

Currie, D. R., Burke, T., Whitney, R. L. & Thompson, D. B. A. 1998 Male and female behaviour and extra-pair paternity in the wheatear. Anim. Behav. 55, 689-703.

Davies, N. B., Butchart, S. H. M., Burke, T. A., Chaline, N. & Stewart, I. R. K. 2003 Reed warblers guard against cuckoos and cuckoldry. Anim. Behav. 65, 285-295.

Delmore, K. E., Kleven, O., Laskemoen, T., Crowe, S. A., Lifjeld, J. T. & Robertson, R. J. 2008 Sex allocation and parental quality in tree swallows. Behav. Ecol. 19, 1243-1249.

Evans, M. L., Stutchbury, B. J. M. & Woolfenden, B. E. 2008 Off-territory forays and genetic mating system of the wood thrush (*Hylocichla mustelina*). Auk 125, 67-75.

Evans, M. L., Woolfenden, B. E., Friesen, L. & Stutchbury, B. J. M. 2009 Variation in the extra-pair mating systems of acadian flycatchers and wood thrushes in forest fragments in southern Ontario. J. Field Ornithol. 80, 146-153.

Freeman-Gallant, C. R. 1996 DNA fingerprinting reveals female preference for male parental care in savannah sparrows. Proc. R. Soc. B 263, 157-160.

Freeman-Gallant, C. R., Wheelwright, N. T., Meiklejohn, K. E., States, S. L. & Sollecito, S. V. 2005 Little effect of extrapair paternity on the opportunity for sexual selection in savannah sparrows (*Passerculus sandwichensis*). Evolution 59, 422-430.

Garvin, J. C., Abroe, B., Pedersen, M. C., Dunn, P. O. & Whittingham, L. A. 2006 Immune response of nestling warblers varies with extra-pair paternity and temperature. Mol. Ecol. 15, 3833-3840.

Gissing, G. J., Crease, T. J. & Middleton, A. L. A. 1998 Extrapair paternity associated with renesting in the American goldfinch. Auk 115, 230-234.

Gyllensten, U. B., Jakobsson, S. & Temrin, H. 1990 No evidence for illegitimate young in monogamous and polygynous warblers. Nature 343, 168-170.

Hatchwell, B. J., Ross, D. J., Chaline, N., Fowlie, M. K. & Burke, T. 2002 Parentage in the cooperative breeding system of long-tailed tits, *Aegithalos caudatus*. Anim. Behav. 64, 55-63.

Hoi-Leitner, M., Hoi, H., Romero-Pujante, M. & Valera, F. 1999 Female extra-pair behaviour and environmental quality in the serin (Serinus serinus): a test of the 'constrained female hypothesis'. Proc. R. Soc. B 266, 1021-1026.

Johannessen, L. E., Slagsvold, T., Hansen, B. T. & Lifjeld, J. T. 2005 Manipulation of male quality in wild tits: effects on paternity loss. Behav. Ecol. 16, 747-754.

Johnsen, A. & Lifjeld, J. T. 2003 Ecological constraints on extra-pair paternity in the bluethroat. Oecologia 136, 476-483.

Klatt, P. H., Stutchbury, B. J. M. & Evans, M. L. 2008 Incubation feeding by male scarlet tanagers: a mate removal experiment. J. Field Ornithol. 79, 1-10.

Kleven, O., Bjerke, B.-A. & Lifjeld, J. T. 2008 Genetic monogamy in the common crossbill (*Loxia curvirostra*). J. Ornithol. 149, 651-654.

Kleven, O., Jacobsen, F., Robertson, R. J. & Lifjeld, J. T. 2005 Extrapair mating among relatives in the barn swallow: a role for kin selection? Biol. Lett. 1, 389-392.

Kleven, O. & Lifjeld, J. T. 2005 No evidence for increased offspring heterozygosity from extrapair mating in the reed bunting (*Emberiza schoeniclus*). Behav. Ecol. 16, 561-565.

Kleven, O., Øigarden, T., Foyn, B. E., Moksnes, A., Røskaft, E., Rudolfsen, G., Stokke, B. G. & Lifjeld, J. T. 2007 Low frequency of extrapair paternity in the common redstart (*Phoenicurus phoenicurus*). J. Ornithol. 148, 373-378.

Krokene, C. & Lifjeld, J. T. 2000 Variation in the frequency of extra-pair paternity in birds: A comparison of an island and a mainland population of blue tits. Behaviour 137, 1317-1330.

Kudernatsch, D., Buchman, M., Fiedler, W. & Segelbacher, G. 2010 Extrapair paternity in a German population of the Northern Wheatear (*Oenanthe oenanthe*). J. Ornithol. 151, 491-498.

Leisler, B. & Wink, M. 2000 Frequencies of multiple paternity in three *Acrocephalus* species (Aves Sylviidae) with different mating systems (*A. palustris*, *A. arundinaceus*, *A. paludicola*). Ethol. Ecol. Evol. 12, 237-249.

Lens, L., Van Dongen, S., Van den Broeck, M., Van Broeckhoven, C. & Dhondt, A. A. 1997 Why female crested tits copulate repeatedly with the same partner: Evidence for the mate assessment hypothesis. Behav. Ecol. 8, 87-91.

Lifjeld, J. T., Slagsvold, T. & Lampe, H. M. 1991 Low frequency of extra-pair paternity in pied flycatchers revealed by DNA fingerprinting. Behav. Ecol. Sociobiol. 29, 95-101.

Meek, S. B., Robertson, R. J. & Boag, P. T. 1994 Extrapair paternity and intraspecific brood parasitism in eastern bluebirds revealed by DNA fingerprinting. Auk 111, 739-744.

Mennill, D. J., Ramsay, S. M., Boag, P. T. & Ratcliffe, L. M. 2004 Patterns of extrapair mating in relation to male dominance status and female nest placement in black-capped chickadees. Behav. Ecol. 15, 757-765.

Morton, E. S., Stutchbury, B. J. M., Howlett, J. S. & Piper, W. H. 1998 Genetic monogamy in blue-headed vireos and a comparison with a sympatric vireo with extrapair paternity. Behav. Ecol. 9, 515-524.

Nadvornik, P. 2004 Application of molecular methods for population studies of selected songbird species. Ph.D. thesis. Olomouc: Palacky University.

Øigarden, T., Borge, T. & Lifjeld, J. T. 2010 Extrapair paternity and genetic diversity: the white-throated dipper *Cinclus cinclus*. J. Avian Biol.48, 248-257.

Olsen, B. J., Greenberg, R., Fleischer, R. C. & Walters, J. R. 2008 Extrapair paternity in the swamp sparrow, *Melospiza georgiana*: male access or female preference? Behav. Ecol. Sociobiol. 63, 285-294.

Orell, M., Rytkonen, S., Launonen, V., Welling, P., Koivula, K., Kumpulainen, K. & Bachmann, L. 1997 Low frequency extra-pair paternity in the willow tit *Parus montanus* as revealed by DNA fingerprinting. Ibis 139, 562-566.

Otter, K., Ratcliffe, L., Michaud, D. & Boag, P. T. 1998 Do female black-capped chickadees prefer high-ranking males as extra-pair partners? Behav. Ecol. Sociobiol. 43, 25-36.

Reudink, M. W., Marra, P. P., Kyser, T. K., Boag, P. T., Langin, K. M. & Ratcliffe, L. M. 2009 Non-breeding season events influence sexual selection in a long-distance migratory bird. Proc. R. Soc. B 276, 1619-1626.

Roberts, P. K. 2005 Determinants of reproductive success in a Neotropoical migratory songbird: timing, site-selection and mating strategies. Ph.D. thesis. Hanover: Dartmouth College.

Rowe, K. M. C. & Weatherhead, P. J. 2007 Social and ecological factors affecting paternity allocation in American robins with overlapping broods. Behav. Ecol. Sociobiol. 61, 1283-1291.

Schmoll, T., Dietrich, V., Winkel, W., Epplen, J. T., Schurr, F. & Lubjuhn, T. 2005 Paternal genetic effects on offspring fitness are context dependent within the extrapair mating system of a socially monogamous passerine. Evolution 59, 645-657.

Segelbacher, G., Kabisch, D., Stauss, M. & Tomiuk, J. 2005 Extra-pair young despite strong pair bonds in the European nuthatch (*Sitta europaea*). J. Ornithol. 146, 99-102.

Sheldon, B. C. & Burke, T. 1994 Copulation behavior and paternity in the chaffinch. Behav. Ecol. Sociobiol. 34, 149-156.

Stapleton, M. K., Kleven, O., Lifjeld, J. T. & Robertson, R. J. 2007 Female tree swallows (*Tachycineta bicolor*) increase offspring heterozygosity through extrapair mating. Behav. Ecol. Sociobiol. 161, 1725-1733.

Stewart, I. R. K., Hanschu, R. D., Burke, T. & Westneat, D. F. 2006 Tests of ecological, phenotypic, and genetic correlates of extra-pair paternity in the house sparrow. Condor 108, 399-413.

Sundberg, J. & Dixon, A. 1996 Old, colourful male yellowhammers, *Emberiza citrinella*, benefit from extra-pair copulations. Anim. Behav. 52, 113-122.

Tarof, S. A., Ratcliffe, L. M., Kasumovic, M. M. & Boag, P. T. 2005 Are least flycatcher (*Empidonax minimus*) clusters hidden leks? Behav. Ecol. 16, 207-217.

Thusius, K. J., Dunn, P. O., Peterson, K. A. & Whittingham, L. A. 2001 Extrapair paternity is influenced by breeding synchrony and density in the common yellowthroat. Behav. Ecol. 12, 633-639.

Vallender, R., Friesen, V. L. & Robertson, R. J. 2007 Paternity and performance of golden-winged warblers (*Vermivora chrysoptera*) and golden-winged X blue-winged warbler (*V. pinus*) hybrids at the leading edge of a hybrid zone. Behav. Ecol. Sociobiol. 61, 1797-1807.

Weatherhead, P. J. & Boag, P. T. 1995 Pair and extra-pair mating success relative to male quality in red-winged blackbirds. Behav. Ecol. Sociobiol. 37, 81-91.

Westneat, D. F. 1990 Genetic parentage in the indigo bunting - a study using DNA fingerprinting. Behav. Ecol. Sociobiol. 27, 67-76.

Wetton, J. H. & Parkin, D. T. 1991 An association between fertility and cuckoldry in the house sparrow, *Passer domesticus*. Proc. R. Soc. B 245, 227-233.

Whittingham, L. A. & Lifjeld, J. T. 1995 High paternal investment in unrelated young - extra-pair paternity and male parental care in house martins. Behav. Ecol. Sociobiol. 37, 103-108.

Yezerinac, S. M., Weatherhead, P. J. & Boag, P. T. 1995 Extra-pair paternity and the opportunity for sexual selection in a socially monogamous bird (*Dendroica petechia)*. Behav. Ecol. Sociobiol. 37, 179-188.
